# A Comparative Analysis of Mammography Uptake between Migrant and Non-Migrant Women in Austria—Results of the Austrian Health Interview Survey

**DOI:** 10.3390/healthcare12151468

**Published:** 2024-07-23

**Authors:** Diana Wahidie, Yüce Yilmaz-Aslan, Patrick Brzoska

**Affiliations:** Health Services Research, Faculty of Health, School of Medicine, Witten/Herdecke University, 58455 Witten, Germany; yuece.yilmaz-aslan@uni-wh.de (Y.Y.-A.); patrick.brzoska@uni-wh.de (P.B.)

**Keywords:** mammography, breast cancer, migrant, heterogeneity, screening, Austria, survey, utilization

## Abstract

Mammography can reduce breast cancer incidence and mortality. Studies on the utilization of mammography among migrant and non-migrant women are inconsistent. Many of these studies do not take the heterogeneity of migrants in terms of ethnicity and country of origin into account. The aim of the present study was to examine disparities in the use of mammography between non-migrant women and the five largest migrant groups in Austria. The study used data from a nationwide population-based survey of 5118 women aged 45 years and older and analyzed the participation in mammography as a dependent variable. Multivariable logistic regression was used to compare mammography uptake between the aforementioned groups of women, while adjusting for socioeconomic and health variables. The study shows that all migrant groups involved tended to use mammography less frequently than non-migrant women; statistically significant differences, however, were only observed for Hungarian migrant women (adjusted OR = 0.36; 95%-CI: 0.13, 0.95; *p* = 0.038) and women from a Yugoslavian successor state (adjusted OR = 0.55; 95%-CI: 0.31, 0.99; *p* = 0.044). These findings are consistent with other studies in Europe and beyond, highlighting the heterogeneity of migrant populations and emphasizing the need for a diversity-sensitive approach to health care.

## 1. Introduction

Breast cancer was estimated to account for 28.7% of all new cancer cases in women in the EU-27 countries in 2020 (this is the most recent data available), making it the most common form of cancer in the female population [1]. Breast cancer incidence rates are mainly rising in the EU-27-countries, which can be attributed to reproductive factors, increasing obesity and physical inactivity, amongst others. In contrast, mortality rates are declining, mainly due to measures for the early detection of breast cancer [1]. Breast cancer screening programs are well established in most European countries, varying between three-year and two-year screening programs in age groups from 45 to 74 years, as this age group has the highest risk of developing breast cancer [2]. Several examination options are available for early detection. These include self-examination of the breast, clinical breast examination by a physician, ultrasound examination, magnetic resonance imaging, and mammography. The only method recognized as effective for detecting precancerous breast lesions is mammography which is the standard approach usually used in screening programs. 

Non-participation in breast cancer screening is associated with older age, low household income, unemployment, and being unmarried [3]. In addition, studies from various European countries show that migrant women are less likely to participate in breast cancer screening programs than the majority population [3,4,5]. For example, a study from Denmark showed that non-Western migrant women were 19% less likely to participate in mammography screening compared to Danish-born women [4]. Similarly, survey data from Spain show that native-born women are about three times more likely to undergo mammography compared to migrant women [5]. Comparable results were reported from other European countries such as the Netherlands [6] and Switzerland [7], and from other regions of the world such as the United States [8], England [9], and Canada [10]. In contrast to these studies, studies from Germany are inconsistent, showing either higher or lower rates of breast cancer screening utilization among migrant as compared to non-migrant women, depending on the age groups analyzed. For example, Kaucher et al. show that resettlers are more likely to participate in the mammography screening program than the general German population [11]. Berens et al. also provide evidence of higher breast cancer screening uptake among migrant women, showing that women of Turkish origin between the ages of 50 and 64 are more likely to attend breast cancer screening than non-Turkish women in Germany. For women of Turkish origin aged 65–69 years, however, they show that they participate less frequently in breast cancer screening [12]. One potential reason for the inconsistent findings across different studies may be that usually the heterogeneity of the migrant population in terms of ethnicity and country of origin could not be taken into account.

The potentially lower uptake of mammography by migrant women can be attributed to a number of barriers they face in the healthcare system. These include poor language proficiency, low literacy levels, and a lack of knowledge and awareness about breast cancer screening [13]. Studies also show that the distance to the screening centers plays a role, particularly in case there is a lack of transport or childcare facilities [13,14]. Cultural factors such as the stigmatization of cancer, religious beliefs, and the fatalistic attitude that the cure for breast cancer is in God’s hands can also lead to lower utilization of breast cancer screening [13,14]. 

In Austria, which has a similar history of migration as Germany, 26.4% of the population are migrants, i.e., individuals who have non-Austrian citizenship or who themselves or whose parents were born abroad [15]. Since January 2014, a population-based breast cancer screening program was made available free of charge to all women between the ages of 45 and 69 in Austria as part of preventive health care. In addition, all women between the ages of 45 and 69 receive an invitation to have a mammography every two years. Women between the ages of 40 and 44 and over 70 can also opt-in to participate. With that invitation, each woman can contact radiologists directly to perform the mammogram without the need for a referral [16]. To date, to the best of our knowledge, there are no studies on the uptake of breast cancer screening among migrant women in Austria. 

The aim of the present study was to examine disparities in the use of mammography between non-migrant women and the five largest migrant groups in Austria (individuals with a nationality from or born in a Yugoslav successor state, Turkey, Romania, Hungary, or Germany) by using data from a representative population-based survey. By doing so, we address not only existing limitations about potential disparities in the use of mammography among migrants in Austria, we also add to the existing literature by exploring how potential disparities vary across different countries of origin.

## 2. Materials and Methods

### 2.1. Data

The study is based on data from the “Austrian Health Interview Survey 2019”, which was conducted by Statistics Austria on behalf of the Federal Ministry of Social Affairs, Health, Care, and Consumer Protection and the Federal Health Agency in 2018/2019 following the approach of the European Health Information Survey. The “Austrian Health Interview Survey 2019” is a representative population-based cross-sectional survey providing data on 15,461 randomly selected individuals aged 15 years and older, recruited on the basis of the Central Population Register and spatially stratified according to the 32 health regions of the Austrian Structure Plan. Data were collected using computer-assisted face-to-face and web-based interviews, supplemented by questionnaires completed by the respondents themselves. Respondents were able to participate in the survey voluntarily, and the response rate was 50.5%. For the present study, only female respondents aged 45 years and older were considered, as mammography is recommended from this age onwards in Austria [17]. The final sample studied consisted of *n* = 5118 women.

### 2.2. Study Variables

In the survey, women were asked when they last had a mammography. The response options were: “within the last 12 months”, “1 to less than 2 years ago”, “2 to less than 3 years ago”, 3 years ago or longer”, and “never”. For the present study, this variable was dichotomized to distinguish between women who had a mammography at least once in their lifetime and those who never had one. The outcomes were compared between non-migrants and the five largest migrant groups living in Austria, i.e., individuals with a nationality from or born in a Yugoslav successor state, Turkey, Romania, Hungary, and Germany. Respondents were considered migrants if they either had non-Austrian citizenship or were born outside of Austria. Covariates included were age (5-year age groups), occupational status (employed, not employed), partnership status (living with a partner in the same household vs. not living with one in the same household), household income (quintiles), and education level. The education level was assessed according to the International Standard Classification of Education (ISCED), which we grouped into three categories: low (ISCED 1–2; primary and lower secondary education), medium (ISCED 3–4; upper/post secondary/non-tertiary education), and high (ISCED 5–8; tertiary education). To account for contextual and health differences between population groups, region of residence (province), degree of urbanization (high, medium, and low), presence of chronic diseases (yes, no), and self-rated health status (1 very good to 5 very poor) were also included.

### 2.3. Analysis

To describe the sample, differences between population groups were analyzed with chi-square tests, with the significance level set at *p* < 0.05. Multivariable logistic regression analysis was performed to examine adjusted differences between population groups with respect to mammography. Results are expressed as adjusted odds ratios (aOR) with 95% confidence intervals (95% CI). All analyses were conducted using IBM SPSS Statistics version 26.

## 3. Results

Of the 5118 respondents in the age group 45 and older, 146 were migrants from a Yugoslav successor state, 140 were German migrants, 51 were Turkish migrants, 31 were Romanian migrants, and 28 were Hungarian migrants. On average, migrant women were younger and more likely to live in urban areas. Turkish migrants, in particular, had a lower level of education, were more likely to suffer from chronic diseases, and rated their health status worse than non-migrants. Mammography uptake was lower among all migrant groups than among non-migrants. While 92.9% of non-migrant women had a mammography at least once before the survey, this percentage was lower among Hungarian (78.6%), Romanian (83.9%), Turkish (86.3%), German (87.9%), and women from a successor state of Yugoslavia (89.7%) (Table 1). 

Differences in mammography uptake remained after adjustment for covariates. All migrant groups were much less likely to have had a mammography before the survey than non-migrant women. Compared to non-migrant women, Hungarian migrant women were at 64% (aOR = 0.36; 95%-CI: 0.13–0.95), Romanian migrant women at 58% (aOR = 0.42; 95%-CI: 0.15–1.16), Turkish migrant women at 57% (aOR = 0.43; 95%-CI: 0.18–1.02), migrant women from a successor state of Yugoslavia at 45% (aOR = 0.55; 95%-CI: 0.31–0.99), and German migrant women at 33% (aOR = 0.67; 95%-CI: 0.39–1.16) lower odds of having a mammography before the survey. However, the differences between non-migrant women and German, Turkish, and Romanian migrant women were not statistically significant (*p* > 0.05). Age, education level, net income, the regions of Burgenland, Lower Austria, Vienna, Styria, and Tyrol, and the presence of chronic diseases were significantly associated with mammography utilization (Table 2).

## 4. Discussion

This study used a nationwide population-based cross-sectional survey to examine the utilization of mammography among non-migrant and the five largest migrant groups living in Austria. The results of the study show that all migrant groups involved were less likely to have a mammography compared to non-migrant women, although, however, the differences between non-migrant women and German, Turkish, and Romanian migrant women were not statistically significant. The differences were not due to differences in sociodemographic, health, or regional factors between the population groups. 

The results to some degree confirm the findings of other studies showing lower uptake of mammography among migrant women compared to the majority population [3,4,5,6,7]. Non-participation in mammography can be attributed to different barriers that migrants encounter in the health care system. These include, among others, lower proficiency of the language spoken in the host country as well as the location of services [18]. For example, migrant women in Australia reported not using mammography because the screening centers were far from their homes and they had limited mobility due to their low income [19]. Other studies, for example from Denmark, also indicate that transportation and inconvenient location of screening centers may lead to lower breast cancer screening participation among migrants [4,20].

To reduce language barriers, interpreters and medical professionals with the same cultural background as the target group should be employed in the health centers. This can contribute to greater self-confidence among migrant women and a more patient-centered atmosphere during care and treatment [19]. 

In addition, the unfamiliar structures of the health care system compared to the country of origin, e.g., availability and organization of screening programs, may hinder participation in mammography for migrant women [19]. Additionally, lack of knowledge about breast cancer symptoms, breast cancer risk factors, and prevention strategies can also lead women to not participate in breast cancer screening [21]. 

Other reasons for the lack of uptake of mammography could be the missing awareness of the risk of breast cancer. For example, the aforementioned Australian study showed that Thai women pay little attention to breast cancer screening when they have no relatives diagnosed with breast cancer and therefore believe they are not at any particular risk [19]. Whether this is also applicable to migrants residing in Austria needs to be explored.

In addition, a lack of motivation to undertake preventive measures in general may also result in some migrant women not attending mammography. A systematic review suggests that Eastern European migrants on average have lower motivation for preventive measures and often rely on health care providers for health-related decisions. Therefore, if health care providers do not actively recommend mammography, those migrant women may not see it as a potentially useful preventive measure. The study attributes this to low health-related self-efficacy and external locus of control [22]. Therefore, health care organizations and providers, especially general practitioners, should take a more proactive role in breast cancer and breast cancer prevention education and provide sufficient information to migrant women to increase their awareness of breast cancer risk. Another tool for educating the target group, as suggested by women themselves, could be television [19]. Furthermore, information material on breast cancer screening should be provided in the migrant women’s native language and made available where the target group is present, e.g., in community centers.

Finally, cultural attitudes toward screening, particularly feelings of shame associated with breast exposure [23,24] and fear of screening and a cancer diagnosis, may discourage migrant women from being screened [21].

The strengths of the present study include the generalizability of the data for the Austrian population and the large sample size. One limitation, however, is the small sample sizes of certain migrant groups. These limit the generalizability of our results and underline the exploratory nature of our study. Therefore, the results should be interpreted cautiously and further research with larger samples of migrants is needed to validate the findings. In addition, the data were collected on the basis of respondents’ self-reports and are not based on administrative or routine data. As a result, recall bias may be present as the survey refers to the utilization of health care services over a period of 10 or more years. Another important limitation is that the survey was conducted only in German, which excluded migrant women with poor language skills from participation and underrepresented them in the study. Consequently, disparities in mammography uptake between migrant and non-migrant women were probably underestimated, as migrant women with poor language proficiency in particular are at risk of perceiving barriers in the health care system. Finally, it should be noted that other factors, such as length of stay in the host country, proficiency in the language of the host country, physician recommendations for participation in mammography, and a family history of breast cancer may also influence mammography uptake, but we were unable to take these into account due to lack of data. In addition, we did not examine the frequency of mammography use. Future studies should consider investigating these factors and exploring differences in the frequency of mammography utilization between migrant and non-migrant women. 

## 5. Conclusions

To the authors’ knowledge, this is the first study to examine differences in mammography uptake between non-migrant women and the five largest migrant groups in Austria. The results of the study show that migrant women living in Austria have a lower uptake of mammography compared to non-migrant women. This could be due to barriers that migrant women face in the health care system. These include language and knowledge deficits, unfamiliar health care structures, and cultural beliefs that are not considered in health care. To address these barriers, measures should be taken to make health care providers aware of the needs of migrant women, in addition to the use of interpreters and multilingual staff. This includes cultural competency training for healthcare staff [25]. Additionally, targeted educational interventions in migrant communities should be implemented to raise awareness about breast cancer and increase health literacy with respect to breast cancer screening to enable women to make informed choices about this health care service. For this purpose, for example, trained health educators from migrant communities could be involved [26]. To overcome logistical barriers, policy measures can be taken to improve access to breast cancer screening facilities. This could be facilitated by providing (financial) resources to support screening facilities with flexible opening hours or by creating new facilities in easily reachable locations [25]. However, it should be kept in mind that migrants are a heterogeneous group and that “one-size-fits-all” solutions are not sufficient. 

## Figures and Tables

**Table 1:** Description of the study sample by population group (Austrian Health Interview Survey 2019, women aged 45 and over, n = 5118).

	Population Group								
	Non-Migrants	Migrants from a Yugoslav Successor State	German Migrants	Turkish Migrants	Romanian Migrants	Hungarian Migrants	Other Migrants	*p* Value *	*All Migrants*
*N*	4507	146	140	51	31	28	215		*611*
**Age** (years)								<0.001	
45–49	539 (12.0%)	31 (21.2%)	18 (12.9%)	14 (27.5%)	8 (25.8%)	10 (35.7%)	45 (20.9%)		*126 (20.6%)*
50–54	641 (14.2%)	32 (21.9%)	28 (20.0%)	17 (33.3%)	10 (32.3%)	3 (10.7%)	40 (18.6%)		*130 (21.3%)*
55–59	694 (15.4%)	27 (18.5%)	19 (13.6%)	5 (9.8%)	2 (6.5%)	5 (17.9%)	29 (13.5%)		*87 (14.2%)*
60–64	639 (14.2%)	18 (12.3%)	11 (7.9%)	3 (5.9%)	6 (19.4%)	1 (3.6%)	26 (12.1%)		*65 (10.6%)*
65–69	537 (11.9%)	17 (11.6%)	4 (2.9%)	7 (13.7%)	0 (0.0%)	5 (17.9%)	16 (7.4%)		*49 (8.0%)*
70–74	441 (9.8%)	9 (6.2%)	19 (13.6%)	3 (5.9%)	0 (0.0%)	0 (0.0%)	20 (9.3%)		*51 (8.3%)*
75–79	479 (10.6%)	7 (4.8%)	18 (12.9%)	1 (2.0%)	2 (6.5%)	2 (7.1%)	11 (5.1%)		*41 (6.7%)*
80+	537 (11.9%)	5 (3.4%)	23 (16.4%)	1 (2.0%)	3 (9.7%)	2 (7.1%)	28 (13.0%)		*62 (10.1%)*
**Partnership status**								0.680	
Partner	2779 (61.7%)	95 (65.1%)	84 (60.0%)	35 (68.6%)	18 (58.1%)	15 (53.6%)	125 (58.1%)		*372 (60.9%)*
No Partner	1728 (38.3%)	51 (34.9%)	56 (40.0%)	16 (31.4%)	13 (41.9%)	13 (46.4%)	90 (41.9%)		*239 (39.1%)*
**Occupational status**								0.002	
Employed	1554 (34.5%)	69 (47.3%)	58 (41.4%)	13 (25.5%)	15 (48.4%)	15 (53.6%)	77 (35.8%)		*247 (40.4%)*
Not employed	2953 (65.5%)	77 (52.7%)	82 (58.6%)	38 (74.5%)	16 (51.6%)	13 (46.4%)	138 (64.2%)		*364 (59.6%)*
**Educational level**								<0.001	
Low	2743 (60.9%)	98 (67.1%)	66 (47.1%)	48 (94.1%)	12 (38.7%)	7 (25.0%)	80 (37.2%)		*311 (50.9%)*
Moderate	1360 (30.2%)	40 (27.4%)	49 (35.0%)	3 (5.9%)	13 (41.9%)	11 (39.3%)	73 (34.0%)		*189 (30.9%)*
High	404 (9.0%)	8 (5.5%)	25 (17.9%)	0 (0.0%)	6 (19.4%)	10 (35.7%)	62 (28.8%)		*111 (18.2%)*
**Net equivalent income of respondent’s household**								0.141	
First income quintile group	1367 (30.3%)	53 (36.3%)	41 (29.3%)	17 (33.3%)	10 (32.3%)	11 (39.3%)	77 (35.8%)		*209 (34.2%)*
Second income quintile group	993 (22.0%)	29 (19.9%)	33 (23.6%)	15 (29.4%)	4 (12.9%)	4 (14.3%)	41 (19.1%)		*126 (20.6%)*
Third income quintile group	1009 (22.4%)	31 (21.2%)	24 (17.1%)	7 (13.7%)	10 (32.3%)	7 (25.0%)	48 (22.3%)		*127 (20.8%)*
Fourth income quintile group	676 (15.0%)	27 (18.5%)	19 (13.6%)	9 (17.6%)	4 (12.9%)	4 (14.3%)	36 (16.7%)		*99 (16.2%)*
Fifth income quintile group	462 (10.3%)	6 (4.1%)	23 (16.4%)	3 (5.9%)	3 (9.7%)	2 (7.1%)	13 (6.0%)		*50 (8.2%)*
**Degree of urbanization of place of residence**								<0.001	
High	578 (12.8%)	46 (31.5%)	26 (18.6%)	17 (33.3%)	5 (16.1%)	9 (32.1%)	90 (41.9%)		*193 (31.6%)*
Moderate	1478 (32.8%)	66 (45.2%)	55 (39.3%)	28 (54.9%)	17 (54.8%)	10 (35.7%)	67 (31.2%)		*243 (39.8%)*
Low	2451 (54.4%)	34 (23.3%)	59 (42.1%)	6 (11.8%)	9 (29.0%)	9 (32.1%)	58 (27.0%)		*175 (28.6%)*
**Region (federal state) of residence**								<0.001	
Burgenland	328 (7.3%)	3 (2.1%)	8 (5.7%)	0 (0.0%)	4 (12.9%)	3 (10.7%)	8 (3.7%)		*26 (4.3%)*
Lower Austria	742 (16.5%)	20 (13.7%)	7 (5.0%)	3 (5.9%)	3 (9.7%)	4 (14.3%)	26 (12.1%)		*63 (10.3%)*
Vienna	335 (7.4%)	27 (18.5%)	11 (7.9%)	11 (21.6%)	5 (16.1%)	8 (28.6%)	73 (34.0%)		*135 (22.1%)*
Carinthia	308 (6.8%)	8 (5.5%)	17 (12.1%)	0 (0.0%)	1 (3.2%)	1 (3.6%)	12 (5.6%)		*39 (6.4%)*
Styria	932 (20.7%)	14 (9.6%)	14 (10.0%)	1 (2.0%)	7 (22.6%)	5 (17.9%)	21 (9.8%)		*62 (10.1%)*
Upper Austria	819 (18.2%)	31 (21.2%)	19 (13.6%)	13 (25.5%)	10 (32.3%)	1 (3.6%)	23 (10.7%)		*97 (15.9%)*
Salzburg	272 (6.0%)	14 (9.6%)	14 (10.0%)	4 (7.8%)	0 (0.0%)	1 (3.6%)	10 (4.7%)		*43 (7.0%)*
Tyrol	524 (11.6%)	17 (11.6%)	28 (20.0%)	11 (21.6%)	1 (3.2%)	3 (10.7%)	23 (10.7%)		*83 (13.6%)*
Vorarlberg	247 (5.5%)	12 (8.2%)	22 (15.7%)	8 (15.7%)	0 (0.0%)	2 (7.1%)	19 (8.8%)		*63 (10.3%)*
**Self-rated health (1—“very good” to 5—“very poor”)**								<0.001	
Very good	1120 (24.9%)	17 (11.6%)	39 (27.9%)	4 (7.8%)	4 (12.9%)	7 (25.0%)	44 (20.5%)		*115 (18.8%)*
Good	1774 (39.4%)	53 (36.3%)	56 (40.0%)	13 (25.5%)	14 (45.2%)	13 (46.4%)	90 (41.9%)		*239 (39.1%)*
Medium	1219 (27.0%)	54 (37.0%)	36 (25.7%)	24 (47.1%)	7 (22.6%)	4 (14.3%)	63 (29.3%)		*188 (30.8%)*
Poor	316 (7.0%)	18 (12.3%)	7 (5.0%)	8 (15.7%)	6 (19.4%)	3 (10.7%)	14 (6.5%)		*56 (9.2%)*
Very poor	78 (1.7%)	4 (2.7%)	2 (1.4%)	2 (3.9%)	0 (0.0%)	1 (3.6%)	4 (1.9%)		*13 (2.1%)*
**Presence of chronic disease**								0.052	
Yes	2283 (50.7%)	79 (54.1%)	59 (42.1%)	34 (66.7%)	14 (45.2%)	11 (39.3%)	115 (53.5%)		*312 (51.1%)*
No	2224 (49.3%)	67 (45.9%)	81 (57.9%)	17 (33.3%)	17 (54.8%)	17 (60.7%)	100 (46.5%)		*299 (48.9%)*
**Mammography**								0.001	
Yes	4189 (92.9%)	131 (89.7%)	123 (87.9%)	44 (86.3%)	26 (83.9%)	22 (78.6%)	194 (90.2%)		*540 (88.4%)*
No	318 (7.1%)	15 (10.3%)	17 (12.1%)	7 (13.7%)	5 (16.1%)	6 (21.4%)	21 (9.8%)		*71 (11.6%)*

* *p*-value refers to a chi-square test comparing non-migrants and the six sub-groups of migrants; values in italics represent the combined results for all migrant groups

**Table 2:** Results of the multivariable logistic regression model with utilization of mammography as the dependent variable. Adjusted odds ratios (aOR) and 95% confidence intervals (95%-CI) (Austrian Health Interview Survey 2019, women aged 45 and over, n = 5118).

Independent Variable	aOR	95%-CI		*p*-Value
**Population group (Ref.: Non-migrants)**				
Migrants from a Yugoslav Successor State	0.55	0.31; 0.99		0.044
German Migrants	0.67	0.39; 1.16		0.155
Turkish Migrants	0.43	0.18; 1.02		0.056
Romanian Migrants	0.42	0.15; 1.16		0.095
Hungarian Migrants	0.36	0.13; 0.95		0.038
Other Migrants	0.78	0.48; 1.28		0.329
**Age (Ref.: 45–49 years)**				
50–54 years	2.50	1.71; 3.67		<0.001
55–59 years	3.20	2.12; 4.82		<0.001
60–64 years	4.61	2.77; 7.67		<0.001
65–69 years	4.67	2.70; 8.01		<0.001
70–74 years	3.75	2.16; 6.52		<0.001
75–79 years	2.67	1.60; 4.46		<0.001
80+	1.00	0.64; 1.57		1.000
**Partnership status (Ref.: No partner)**				
Partner	1.17	0.90; 1.53		0.244
**Occupational status (Ref.: Not employed)**				
Employed	1.47	1.00; 2.14		0.048
**Educational level (Ref.: Low)**				
Moderate	1.14	0.87; 1.49		0.336
High	0.61	0.41; 0.89		0.010
**Net equivalent income of respondent’s household (Ref.: First income quintile group)**				
Second income quintile group	1.30	0.96; 1.76		0.087
Third income quintile group	1.36	0.97; 1.89		0.072
Fourth income quintile group	1.79	1.18; 2.70		0.006
Fifth income quintile group	2.07	1.25; 3.44		0.005
**Degree of urbanization of place of residence (Ref.: High)**				
Moderate	0.92	0.56; 1.53		0.755
Low	0.74	0.45; 1.20		0.220
**Region (federal state) of residence (Ref.: Vorarlberg)**				
Burgenland	2.11	1.15; 3.87		0.016
Lower Austria	1.81	1.12; 2.92		0.015
Vienna	2.13	1.02; 4.46		0.045
Carinthia	1.72	0.99; 3.02		0.056
Styria	1.82	1.15; 2.89		0.011
Upper Austria	1.27	0.81; 1.97		0.300
Salzburg	1.70	0.96; 3.02		0.070
Tyrol	1.89	1.15; 3.09		0.012
**Self-rated health (1-“very good” to 5-“very poor”) (Ref.: Very poor)**				
Very good	1.36	0.66; 2.79		0.402
Good	1.47	0.75; 2.91		0.266
Medium	1.94	0.99; 3.78		0.053
Poor	1.84	0.87; 3.87		0.110
**Presence of chronic disease (Ref.: No)**				
Yes	1.67	1.29; 2.17		<0.001

## Data Availability

The data utilized in the present study may be obtained from Statistics Austria at no cost upon reasonable request (https://www.sozialministerium.at/Themen/Gesundheit/Gesundheitssystem/Gesundheitsberichte/%C3%96sterreichische-Gesundheitsbefragung-2014-(ATHIS).html (accessed on 21 July 2024); information is only available in German language).
